# Analysing the link between public transport use and airborne transmission: mobility and contagion in the London underground

**DOI:** 10.1186/s12940-018-0427-5

**Published:** 2018-12-04

**Authors:** Lara Goscé, Anders Johansson

**Affiliations:** 1 University College London, London, UK; 2 University of Bristol, Bristol, UK

**Keywords:** Public transport, Crowd modelling, Underground, Influenza

## Abstract

**Background:**

The transmission of infectious diseases is dependent on the amount and nature of contacts between infectious and healthy individuals. Confined and crowded environments that people visit in their day-to-day life (such as town squares, business districts, transport hubs, etc) can act as hot-spots for spreading disease. In this study we explore the link between the use of public transport and the spread of airborne infections in urban environments.

**Methods:**

We study a large number of journeys on the London Underground, which is known to be particularly crowded at certain times. We use publically available *Oyster card* data (the electronic ticket used for public transport in Greater London), to infer passengers’ routes on the underground network. In order to estimate the spread of a generic airborne disease in each station, we use and extend an analytical microscopic model that was initially designed to study people moving in a corridor.

**Results:**

Comparing our results with influenza-like illnesses (ILI) data collected by Public Health England (PHE) in London boroughs, shows a correlation between the use of public transport and the spread of ILI. Specifically, we show that passengers departing from boroughs with higher ILI rates have higher number of contacts when travelling on the underground. Moreover, by comparing our results with other demographic key factors, we are able to discuss the role that the Underground plays in the spread of airborne infections in the English capital.

**Conclusions:**

Our study suggests a link between public transport use and infectious diseases transmission and encourages further research into that area. Results could be used to inform the development of non-pharmacological interventions that can act on preventing instead of curing infections and are, potentially, more cost-effective.

## Introduction

Epidemic outbreaks have always been present throughout human history, affecting peoples day-to-day life. Having an improved knowledge of infectious disease spreading mechanisms and control measures is pivotal for humanity’s well-being. Furthermore, gaining a better understanding of how interactions between infective and healthy individuals lead to contagion is a crucial step in that direction. The idea behind our work arose from the necessity of creating more realistic models describing the spread of infectious diseases that could be used for policy making in order to have an impact on a population’s daily life. As an example, in the specific case of influenza, UK Department of Health, created the 2011 *UK Influenza Pandemic Preparedness Strategy* [1] where effective response to a pandemic are set out. The document reports that there is not enough evidence that restrictions on mass gatherings will have any significant effect on influenza virus transmission. Furthermore, from the literature and available data, there is no conclusive evidence of the individual effect of restrictions of mass gatherings to help reduce influenza transmission. As a consequence, the UK Government’s position on large public gatherings, crowded events travelling and public transport use is not only neutral in light of lack of evidences, but those types of events are even encouraged because they represent an important indicator of ’normality’ and may help maintain public morale during a pandemic [1].

When studying the spread of airborne infections on a metropolitan scale there are some environments that serve as seeds of epidemic spread, where a higher number of individuals get in contact with each other. Previous work [2] has shown that incorporating elements of pedestrian modelling [3–9]) when studying the spread of a generic airborne infection in crowded environments, can greatly improve the model’s fidelity. Combining elements of traditional compartmental models [10, 11], network models [12, 13] and wearable sensor based studies [14–16], an analytical method was recently devised [2] with the aim to be able to more accurately model small scale scenarios (i.e. pedestrian interaction). Here we apply this mathematical description to the particular case of the London Underground network by inferring a passenger transport model from data obtained from the TfL (Transport for London) [17].

Since it opened in 1863, the London Underground has become the most important transport network of the English capital and is considered the oldest rapid transit system in the world. It serves 270 stations, has 402 km of extension and carries a number of 1.265 billion annual passengers. Therefore, its stations constitute an ideal use case of crowded and confined environments and can be analysed while studying crowd dynamics and contagion mechanisms.

In the first part of our work we mathematically derive, from the TfL dataset, the time individuals take to move in a system of two stations connected with each other. Furthermore, we show how this model can be extended to a whole line of the London Underground and, from this, we evaluate the number of contacts and new infections in some selected stations. In the second part, we use real data on influenza-like-illnesses (ILI) collected by NHS from GPs in London boroughs and show the correlations between the use of the underground and new ILI infections.

## Method

Previous work [2] has studied the number of new infections created when infective individuals (along with susceptible ones) move in a corridor. Instead of completely modifying the compartmental model framework, the attention was focused on the transmission rate that is generally considered constantly equal to *λ*=*cβ* in the traditional approach, where *β* is the contact rate and *c* represents the proportion of contacts that end up becoming infected. In the new definition fully explored in [2] it is shown how, from an individual-level perspective, the contact rate *β* can be derived from the average local density around the infective individual 〈*ρ*〉, which can be estimated statistically from the average density within a larger space *ϱ*, using first an Eulerian description of the crowd density Gamma distribution $p^{s}(\rho ;A;B)=\frac {B^{A}}{\Gamma (A)}\rho ^{A-1} e^{-B\rho }$ where *A*=3*μ*=3*ϱ*=3*N*/(*Lw*) and then re-formulated into a Lagrangian description $p^{t}(\rho ; A; B) = \frac {p^{s}(\rho ; A; B)}{\langle v(\rho)\rangle }$. Since $0.05 \le \langle v(\rho)\rangle = \frac {1/\sqrt {\rho } - 1/\sqrt {\rho _{\text {max}}}}{0.5} \le 1.34$ is the average velocity of a pedestrian, the average local density around an individual can be formulated as:

1 $$ \langle \rho \rangle = \int_{0}^{\infty} \ p^{t}(\rho)\rho\, d\rho   $$

Moreover, when breathing, large droplets are carried up to 1 meter (2 meters when coughing and 6 meters when sneezing) [18–20]. Susceptible individuals can become infected by inhaling these droplets thus, the density-dependent transmission rate can be re-defined as:

2 $$ \lambda^{\text{density-dependent}} = c \langle \rho \rangle \pi R^{2}   $$

where *πR*^2^ is the area that surrounds the infective individual, according to a radius *R* meters which represents the maximum distance large droplets can be carried to. Consequently, in order to calculate the number of infections produced by an individual walking in a corridor, we need to calculate the average density of the whole corridor.

When considering the path that individuals walk inside a station, we can visualise it as a corridor from the entrance of the station to the train platform (and vice versa). If we can estimate the average density inside that station, at any time of the day, we can infer the local density and, consequently, the transmission rate, thus obtaining the number of new infections occurring in that station throughout the day. We know that the average velocity of individuals moving in the station is *v*=*D*/*T*, where *D* is the distance walked by the individual from the entrance to the train (or from the train to the exit) within a duration of *T* seconds. Moreover the average velocity as $v(\varrho) = \frac {\frac {1}{\sqrt {\varrho }} - \frac {1}{\sqrt {\varrho _{\text {max}}}}}{0.5}$ is derived in [4] from a microscopic model of pedestrian flow, and is defined considering the difference between the net distance between individuals and the mean distance between their center of masses over the net-time headway, and where *ϱ*_*max*_ is the maximum value of the density. Thus, we can infer the average density *ϱ* by knowing the time *T* the individuals take to walk inside the station having *v*(*ϱ*)=*D*/*T* and obtaining

3 $$ \varrho = \frac{4 \varrho_{max}}{\left(\frac{D}{T} \sqrt{\varrho_{max}}+2\right)^{2}}  $$

This means that, in order to solve the epidemiological problem of the number of new infections occurring in the stations of the network, we need to initially solve a pedestrian dynamics model allowing us to determine the time individuals take to walk inside the station at different times of the day.

In the first week of November 2009, TfL collected data from the *Oyster cards* (the electronic ticket used on public transport in Greater London) and made around 10% of them available to the public. When people use the underground, they tap their Oyster card once at the entrance and once again at the exit. These journeys were sampled randomly and the time stamps and Oyster card ID is reported for each entry and exit. TfL also provides a 100% sample of the total average number of entries to and exits from all the stations, every fifteen minutes. Using these data sets, we develop a method to infer the time a passengers take to walk across their starting and arrival stations. By calling *A* the starting station of a generic journey and *B* the arrival station, we can consider the total journey time as a sum of smaller trips.

The total journey time for a single passenger *ΔT*_*A*−>*B*_=

the time required to walk across the starting station from the entrance to the platform $\Delta t^{A}_{en}$ +

+ the waiting time on the platform $\Delta t^{A}_{P} $

+ the travel time the train takes to go from *A* to *B*, *Δt*_*A*−>*B*_ (for simplicity we do not consider trips that require a change of line)

+ the time to walk across the arrival station from the platform to the exit $\Delta t^{B}_{ex}$.

4 $$ \Delta T_{A->B} = \Delta t^{A}_{en} + \Delta t^{A}_{P} + \Delta t_{A->B} + \Delta t^{B}_{ex}   $$

Our goal is to find an estimation of the average density in the station *ϱ*=*N*/*Lw* (where *L* and *w* represent respectively length and width of the walked path). Since this number is clearly not constant during the day (from the station opening until its closure) because the number of people using the station *N* varies throughout the day, we study the average density in a time interval of 15 min and reiterate the process throughout the day. In order to do so we look at the data of the journeys in that specific time interval.

The Oyster card provide only enough data to infer *ΔT*_*A*−>*B*_, while the remaining terms of Eq. (4) are unknown. To solve this problem we use data collected by a train tracking system (developed at CASA [21]) which reports the exact position of each train of the network every 3 min. By knowing the positions of all the trains in the network we are able to infer the time the train takes to move from one station to the next, and the average waiting time at the platform $\left (\Delta t^{A}_{P} + \Delta t_{A->B}\right)$. The still analytically unsolvable equation can now be solved computationally using the method of least squares to calculate the walking time across each of the two stations.

**Fig. 1:**
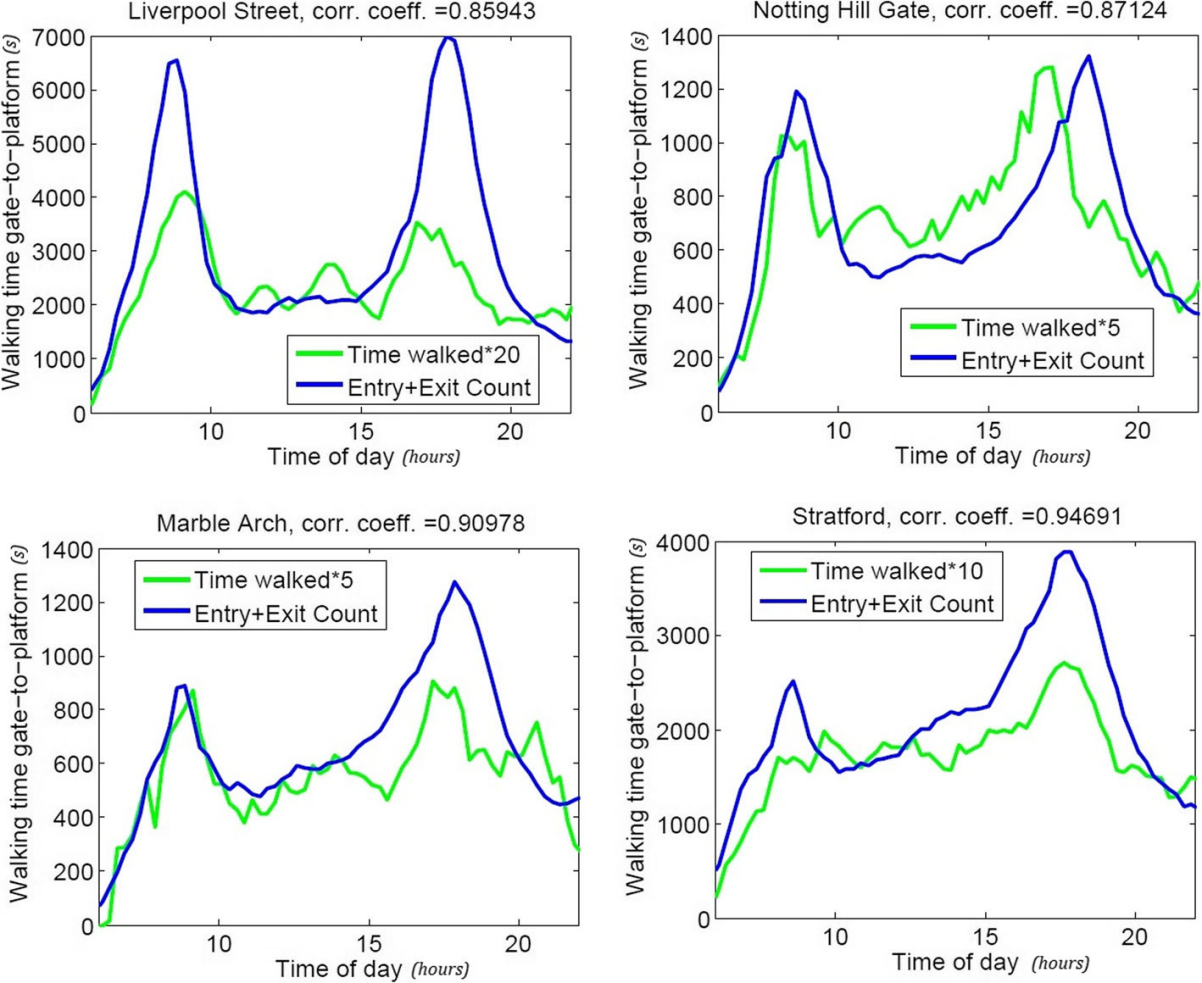
Correlation coefficients for four stations on the Central line. We analysed all Central line trips arriving and departing in a given station (Liverpool Street n=14723; Notting Hill Gate n=5058; Marble Arch n=4102; Stratford n=8426). Results show a peak configuration highlighting the fact that people take more time to traverse the stations during specific times of the day. The correlation coefficient between the time necessary to walk from the entrance of the station to the platform (and viceversa) and the maximum number of individuals in the station at a specific time (data provided by TfL on the number of people entering and leaving each stations every fifteen minutes) show that these times are strictly connected to the density in the station, meaning that more crowded is an area the longer it will take to traverse it. The walking times are multiplied by 5, 10 or 20 for display purposes

As an example, we show here the solutions obtained when studying the Central Line (49 stations and 74km of extension). For practical reasons we show here curves for only a few of the involved stations (Fig. 1) and report in Table 1 the correlation coefficients for the other Central line stations.

**Table 1:** Correlation Coefficients between the times required to traverse the station and the cumulative number of passengers entering and exiting the station in a 15-min period

Stations	Correlation coefficient	Total number of trips (n)
Bank	0.81936	8092
Barkingside	0.52114	466
Bethnal Green	0.87188	5587
Bond Street	0.83662	9705
Buckhurst Hill	0.71433	949
Chancery Lane	0.72835	5591
Debden	0.72102	917
East Acton	0.67666	1625
Gants Hill	0.73208	2272
Greenford	0.64114	1435
Hanger Lane	0.84476	1302
Holborn	0.89477	9414
Holland Park	0.83606	1394
Lancaster Gate	0.75767	1604
Leyton	0.7258	4718
Leytonstone	0.80104	4235
Liverpool Street	0.85943	14723
Loughton	0.52744	1265
Marble Arch	0.90978	4102
Mile End	0.82994	5501
Newbury Park	0.72508	1655
North Acton	0.69053	864
Notting Hill Gate	0.87124	5058
Oxford Circus	0.91423	16400
Perivale	0.8203	1028
Queensway	0.8781	2069
Redbridge	0.7127	1026
Ruislip Gardens	0.68847	422
Snaresbrook	0.44928	1124
South Ruislip	0.66625	764
South Woodford	0.377	1643
St. Paul’s	0.88929	5408
Stratford	0.94691	8426
Tottenham Court Road	0.90168	7985
Wanstead	0.78013	944
White City	0.82213	2953
Woodford	0.80346	2297

Results are shown for most of the Central Line stations (*μ*=0.76178,*σ*=0.13204). Since TfL provides a 10% sample of travels happening during a single week, of which we analyse only the ones happening during weekdays and not involving a change of line, data points for some of the less busy stations were not available or available only during peak times translating into a lower correlation coefficient. In general, a higher amount of data points ensures a more accurate correlation coefficient value. Note that we have excluded stations that had less than *N* trips in any one 15-minute segment, where *N* is the average number of trips (i.e. passengers) per station given by ratio between the total number of trips departing(arriving) from the selected station and arriving(departing) in one of the other stations on the line, over the number of station in that line

Figure 1 highlights two important consequences arising by the use of this method: *(i)* the presence of peaks and *(ii)* the correlation between the curves.

Firstly, the model is able to capture the expected bi-modal behaviour: the morning and the afternoon peaks, meaning that, at the times when the stations are more crowded i.e. around 9 am and 6 pm when people travel to/from work it takes longer to traverse the stations. Moreover by comparing the Time Walked curve (i.e. the curve that represents the time it takes each moment of the day to cross the selected station) with the curve given by the maximum number of individuals present in the station during the day we observe a high correlation coefficient between the two. Consequently, we can say that the method captures the fact that the more crowded a station is, the longer it will take to walk through it.

From the times required to walk inside each stations we can determine the transmission rate defined in Eq. 2. Finally the number of new infections that arise from the contacts happening inside each single station during the whole day can be calculated solving the simple compartmental model described in [2]

5 $$ \begin{array}{rl} {\dot s} =& - \lambda i s \\ {\dot e} =& \lambda i s \\ {\dot i} =& 0 \\ \end{array}   $$

Where *s* and *i* are the compartments representing susceptible and infectious individuals respectively and *e* is the compartment of exposed (infected but not infectious) individuals.

### Influenza-like illnesses

The theoretical description of the model has been presented in relation to a generic airborne human infection. In order to test the applicability of the model in a realistic context, we will now focus our attention on Influenza-like illnesses (ILI). ILI is described by the Centers for Disease Control and Prevention (CDC) as a nonspecific respiratory illness characterized by fever, fatigue, cough, and other symptoms. The majority of ILI cases is not caused by influenza viruses but by others such as rhinoviruses and respiratory syncytial virus (RSV) adenoviruses, and parainfluenza viruses. ILI infections can lead to serious complications and require hospitalization. Moreover individuals can average one to three (adults) and three to six (children) ILI yearly [22]. Table 2 reports data collected by Public Health England (PHE) [23] of the average rate per 100,000 practice population of ILI cases observed from October 2013 until March 2014 in each London borough. The data from PHE were obtained by a large surveillance system that monitors in hours general practitioners (GPs) consultations for a number of key clinical indicators. In United Kingdom individuals register with a single primary care physician who has a well defined patient population. On a daily basis this system reports and covers over 40% of the England population. Data were collected from available practices located in each London borough from October 2013 to March 2014, Table 2 reports their average value during these six months.

**Table 2:** In the second column rate per 100,000 practice population of observed number of ILI cases from October 2013 until March 2014 for each London borough (n=32) are shown

Borough	Rate of observed cases	*Φ*-values
Barking	13.65	2.3819
Barnet	10.35	1.0831
Bexley	5	No Underground
Brent	15.18	1.2586
Bromley	5.96	No Underground
Camden	12.00	1.2365
Croydon	9.64	No Underground
Ealing	7.72	0.9672
Enfield	10.81	1.5157
Greenwich	17.23	8.7555
Hackney	13.16	1.042
Hammersmith and Fulham	1.98	1.2096
Haringey	7.73	3.2414
Harrow	16.98	0.7509
Havering	1.02	1.0846
Hillingdon	9.87	0.2961
Hounslow	1.00	1.3454
Islington	15.37	2.0261
Kensington and Chelsea	5.5	1.161
Kingston upon Thames	4.9	No Underground
Lambeth	12.84	4.3647
Lewisham	11.75	No Underground
Merton	8.41	2.1899
Newham	15.67	4.7831
Redbridge	5.54	1.0542
Richmond upon Thames	2.3	1.8118
Southwark	16.83	4.4972
Sutton	8.40	No Underground
Tower Hamlets	16.66	2.2178
Waltham Forest	10.35	4.7722
Wandsworth	11.04	3.3296
Westminster	6.96	0.8579

In the third column, each borough *Φ*-values are presented. A correlation coefficient of 0.44 is obtained

In order to investigate the correlation between ILI rates in London and the contacts arising when using the underground, we define two additional parameters: *(i)* the total number of contacts occurring for a single passenger during their whole trip *Ψ*, *(ii)* and the total number of contacts occurring for all passengers departing from the same borough in the same time interval during the duration their trips *Φ*.

The quantity *Ψ*, defined as the sum of all the people each passenger get in contact with during their trip, is $\Psi = \sum _{i=1}^{n} {\delta }_{i}$ where *n* is the number of stations the passenger need to cross during the trip and *δ*_*i*_ is the number of contact the passenger makes in the *i*-th station and by our model is equal to

6 $$ \delta_{i} = \langle \rho_{i} \rangle \pi R^{2}  $$

where 〈*ρ*_*i*_〉 is the average density in the *i*-th station.

While comparing data and results we need to keep in mind that our model is applied to the very early stages of contagion in environments considerably smaller than the usual scale (a whole city, or even a nation). To be able to perfectly compare the results from our microscopic analysis we would need individual-level data that are very difficult to acquire, thus in the comparison with PHE data (that are population-level data) we need to take into account the inferential fallacy that may occur when statistical properties observed on an aggregate level do not reflect the relations that exist on a local level. In the attempt of overcoming this problem we study the amount of contacts obtained during the whole trips when leaving from stations belonging to the same boroughs. This translate by saying that for each borough we calculate the number *Φ* that is given by summing for all the trips departing in each underground station of the borough between 5 am and 10 am, the total amount of contacts acquired during each whole trip. Normalised by the total number of people entering the stations of the borough (thus, consequently, the number of departures) between 5 am and 10 am (*N*_*entry*_).

7 $$ \Phi = \cfrac{\sum_{n} \Delta_{n} + \sum_{m} \Delta_{m} + \sum_{k} \Delta_{k} + \dots}{N_{entry}}  $$

where *n* is the total number of trips departing from the first station, *m* is the total amount of trips departing from the second station and so on.

## Results

We compared number of contacts calculated using our model with data collected by Public Health England (PHE) of Influenza-like illnesses (ILI) [23] and several interesting results can be highlighted.

First of all, boroughs that do no contain any underground station seem to have incidence rates lower than than average 9.73 (per 100,000). The average ILI incidence in boroughs without underground is 7.61, while it is 10.24 in boroughs with underground station. One exception is Lewisham (11.75) that, however, has a high number of railway stations (London Overground and Docklands Light Railway, DLR), here the 2011 Census [24] reported railway as the principal form of transport that residents of the borough used to travel to work. This difference, however, is not statistically significant (*p*-value = 0.0776).

We also notice that boroughs with higher case rates are generally more peripheral in respect to others, in particular their underground stations have a more peripheral position on the map, meaning that people who travel from there are forced not only to spend more time on the train, but also to change line one or even several times, consequently getting in contact with a higher number of individuals.

As an example we compared trips from two different boroughs: Islington and the Royal Borough of Kensington and Chelsea (RBKC). Both are very central with respect to the London map, but Islington has a case rate of 15.37 and its stations are more peripheral with respect to the underground map (the majority of the stations are served by only one line), while RBKC has a rate of 5.5 and its stations are more central and well connected and generally served by two or more lines. In order to apply our method in relation to the data, we must consider only the trips made by individuals residing in these boroughs. TfL does not provide personal data of the passengers, however not all underground users reside in London (e.g. tourists, commuters etc), moreover passengers can change position multiple times throughout the day. For this reason, we filtered our data by only considering trips with a departure time between 6 am and 10 am assuming that people leaving for work early in the morning tend to use the more convenient public transport available to them by taking the train from the station closest to their residence. After that, we evaluated all the trips leaving from the stations in these boroughs between the selected time interval, and considered the arrival stations, the lines and stations that were involved, and the total journey time. The first noticeable result is that almost all the trips departing from stations in the borough of Islington have to change line in King’s Cross St. Pancreas station which is one of the busiest and most central station of the entire underground, also connected with a major London railway terminal of the same name. Consequently, for each trip we also need to consider the time the individuals take in this station when changing from a platform to another and the number of contacts they make during the process. The majority of trips departing from RBKC are instead direct trips and do not stop at any intermediate station.

**Fig. 2:**
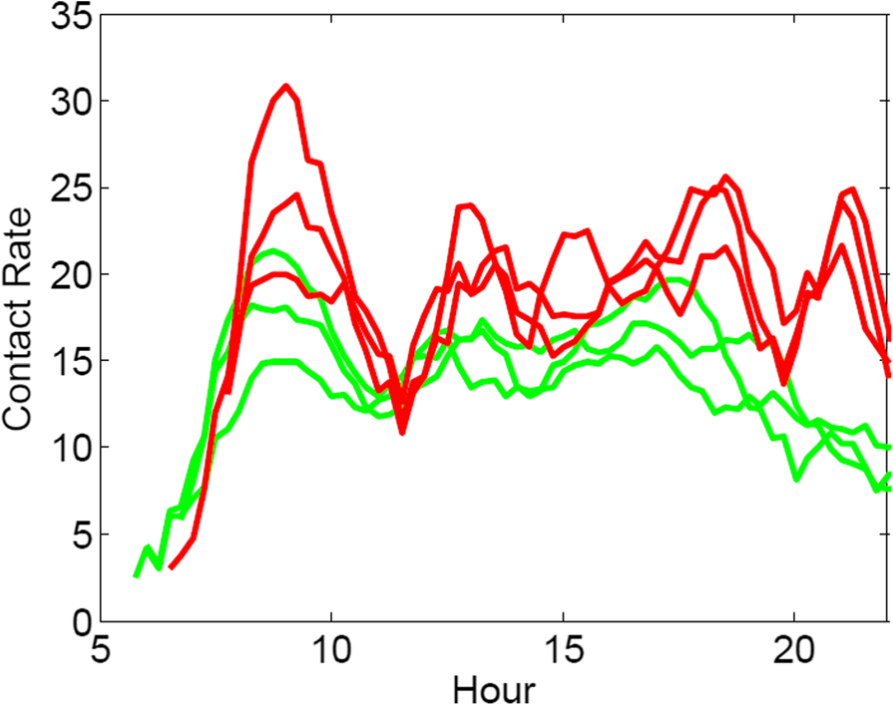
Total number of contacts during trips departing from Islington (red) and during trips departing from RBKC (green). We plotted results for the n=3 most common routes per borough. Passengers leaving from Islington, while travelling on the underground, need to change lines more frequently respect to people departing from RBKC thus traversing more stations and getting in contact with more people

Now, let us evaluate the total number of contacts for several frequent trips departing from stations from both boroughs. Results can be seen in Fig. 2. For trips departing from RBKC most of the time only two stations are involved and therefore

8 $$ \Delta_{RBKC} = \delta_{O} + \delta_{D}  $$

where *δ*_*O*_ is the number of contacts obtained by crossing the origin station, and *δ*_*D*_ is the number of contact resulted by crossing the destination station.

On the other hand, the majority of trips departing from Islington have to stop in an intermediate station in order to change line, most of the times King’s Cross station. Thus, the total number of contact for each trip is calculated by

9 $$ \Delta_{RBKC} = \delta_{O} + \delta_{KC} + \delta_{D}  $$

where *δ*_*KC*_ is the number of contacts made at King’s Cross.

This supports our assumption that individuals leaving from the borough with the highest incidence rate make more contacts with respect of the ones leaving from the borough with the lowest incidence rate.

**Fig. 3:**
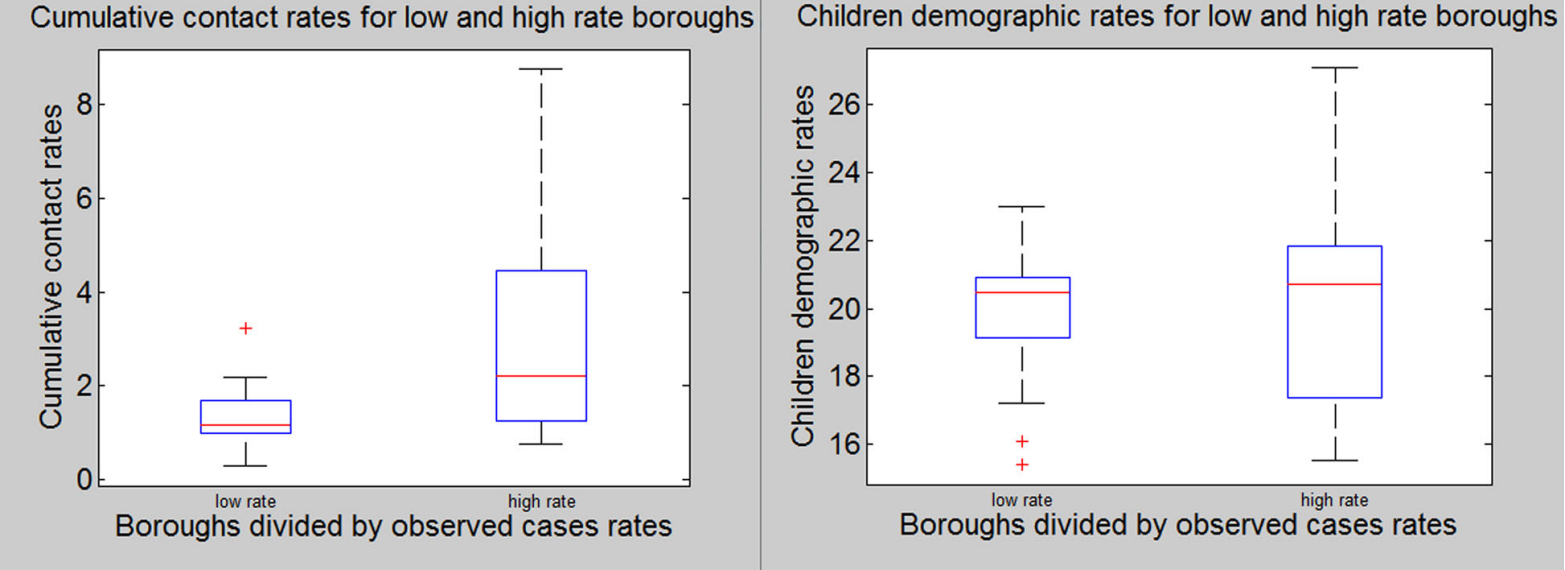
Box Plot Left: Boroughs divided by high (n=15) and low (n=11) incidence rates and their associated *Φ*-values. It is possible to notice a clear difference between the two sets medians (bands inside the boxes). Moreover, high rates boroughs exhibit a considerably higher interquartile range, meaning that cumulative contact rates for boroughs with ILI incidence rates higher than 10 tend to exhibit a significantly higher range variability respect to boroughs with lower ILI incidence rates. This distance, which represents the middle 50% of data, touches considerably higher *Φ*-values for high ILI-rates boroughs respect to the values covered by low ILI-rates boroughs. A Mann-Whitney U-test was run with MATLAB with the null hypothesis of both sets having an equal median and the test rejected the hypothesis with a *p*-value of 0.0293.Right: Boroughs divided by high (n=15) and low (n=11) incidence rates and their associated children demographic rates. It is possible to notice that the difference between the two sets medians (bands inside the boxes) is almost non existent. Moreover, even if high rates boroughs exhibit higher interquartile range, this distance, which represents the middle 50% of data, touches lower children demographic rates for high ILI-rates boroughs respect to the values covered by low ILI-rates boroughs

In order to extend our analysis to the whole city, we use the previously defined parameter *Φ*. The quantity *Φ* has no direct epidemiological meaning, it is just an index of comparison between our model results and PHE data. For this reason, we do not expect to find a perfect correlation between the data and these values, also because PHE data are very aggregate and do not report personal information relating the infective individuals such as sex or age, and we know, for example that everyday commuting across the city is mostly done by adult individuals. Moreover, contact rates would only be able to capture what happens in the underground while PHE data report infection cases independently from the origin of the contagion (may it be in schools, offices, households etc). However, as can be seen from *Φ* values reported in Table 2, this very basic type of comparison can still capture a correlation of 0.44. The model can especially capture what happens in boroughs that have generally higher incidence rates and the respective total amount of contact *Φ* is higher (ex. Greenwich, Newham, Southwark). On the other hand, it is less able to capture what happens in some low incidence boroughs. One explanation is possibly related to the fact that, according to 2011 Census, people living in those boroughs use mostly private means to go to work (car, walk etc) compared to the underground, thus meaning that even though taking the underground from those boroughs involve an average or high amount of contacts, there are actually not many people taking it in the first place (ex. Hounslow, Richmond upon Thames, Merton). Moreover, since the mean incidence rate is 9.73, we divide boroughs into two groups: high incidence rates (≥10) and low incidence rates (<10), and compare the respective *Φ*-values. Results can be seen in Fig. 3. The model is able to capture the fact that the totality of higher incidence stations have an higher amount of contacts and, vice versa, the totality of lower incidence stations have a lower amount of contacts. A Mann-Whitney U-test was run with the null hypothesis of both sets having an equal median and the test rejected the hypothesis with a *p*-value of 0.0293; showing a correlation between the total amount of contacts passengers make when using the underground and infection rates.

This correlation is especially significant if we compare our results with with demographic rates from each borough in Table 3, where we can see that the role of public transport in relation to the spread of ILI could be compared to the role of other important key factors. We collected demographic data by borough from London Datastore [25], ranked them according to their ILI rate and aggregated by comparing boroughs according to the mean ILI incidence of 9.73 (as done previously) and studied the correlation. Some of the results reported in the table confirm a statistical significance for some well-known factors such as boroughs inner densities (p=0.0151), employment rates (p=0.0433), claimants of benefits on housing and income support (p=0.0031) and population aged 65+ (p=0.0012). More surprisingly is the low correlation with the younger population aged <15 years old. Many studies have shown the important role played by children in the spread of infectious diseases because of their higher mixing rates and incidence rates and shown that schools constitute some of the main sites of contagion [26–33]. When comparing PHE data with demographic data on the portion of the population aged 0–15 years old in each borough (knowing that in UK children generally attend the school in their neighbourhood) we find a correlation coefficient of only 0.13. This result seems, at first, particularly surprising, when thinking about the significant role children play in the spread of ILI. Mughini et al. [34] performed a survey analysis on ILI in families with children younger than 4 years, results showed that parents of ILI-affected children had a concurrent 4-fold higher ILI risk. The small statistical difference in children population between boroughs with low and high ILI rates (Fig. 3), however, does not mean that children do not contribute to the spread of ILI on a population level, on the contrary many factors are involved and the larger the scale of the analysis and grater is the amount of time passed since contagion, more complicated is the understanding of what the cause was. The scope of this analysis is simply to compare some factors whose role in the spread of infections is already acknowledged and discuss the possibility that the use of public transport may play a similar role and should be taken into account along them.

**Table 3:** Correlation coefficients between the rates of observed ILI cases and some 2015 demographic data for each borough from London Datastore [25]

Rates	Correlation coefficients	*p*-value
Underground related contacts	0.44	0.0293
Population size	0.3381	0.0676
Inner densities	0.41	0.0151
Employment rates	-0.44	0.0433
Employment with degree	-0.08	1.0000
Benefits claimants	0.54	0.0031
Cars per households	-0.43	0.0103
Population aged 0–15 years old	0.13	0.8504
Population aged 65+	-0.5782	0.0012

Since the mean incidence rate is 9.73, we divided boroughs into two groups: high incidence rates (≥10, *n*=15)) and low incidence rates (<10, *n*=11) and demographic data were divided accordingly. Mann-Whitney U-test was run for all of them with the null hypothesis of both sets having an equal median. It can be seen that, in relation to the spread of infectious diseases, the use of public transport can possibly play a role comparable to the one played by some key factors such as inner densities and employment rates and population by age

## Discussion

The correlation between the use of public transport and the spread of infectious diseases is something that has always been assumed and generally accepted but has never been proved.

Previous studies have highlighted the importance of analysing social contacts and mixing patterns when studying infectious diseases transmitted by the respiratory or close-contact routes. In [26] data were collected by a population-based survey of mixing patterns in eight European countries through a paper-diary methodology. The definition of contact used in the study was of interactions such as a kiss or a handshake for physical contacts, while nonphysical contacts were situations such as a two-way conversation without skin-to-skin contact. In the model that we use [2], instead, we define two individuals as in contact if the local density around them is high enough that they enter in the respective infective regions that surrounds them. Another type of survey based study [35] collected data of ILI cases across UK population through Flusurvey, an internet-based open community cohort. They calculated ILI incidence week by week throughout five months, and investigated possible risk factors associated with it. One of the main conclusions of this study was that public transport does not increase the possibility of acquiring ILI. However, that study took the whole UK into account and the survey approach implied that people were able to report their symptoms at any point throughout their convalescence, moreover symptoms usually arise awhile (even days) after the contagion happens, meaning that people using public transport and people not using it have enough time to mix with each other in other environments (offices, households, etc). Consequently it is not possible to pinpoint exactly where the contagion took place, giving the macroscopic result of no connection between public transport use and incidence of new infection, while a more detailed microscopic analysis would be needed. In our work, instead of taking the whole of UK into account, we focus the attention on a local description where the underground stations are seen as confined and crowded spaces. We study the infection processes on the very first moments of the contagion, actually limiting our study to 15 min segments of intervals, knowing that the mixing patterns that arise once outside the underground network (households, offices etc) will lead to new infections that can blur the public transportation role when looking at the bigger picture. Also, studying the connection between public transport and the spread of airborne infections can highlight another issue i.e. the spatial incidence patterns over time, meaning that it could be possible to find out whether London Underground contribute to how quickly and how strongly a disease spreads to different areas of the city, areas that maybe otherwise would be reached by the disease more slowly or with lower case incidence.

The hypothesis of an association between public transport and disease transmission is not novel. Another study [36] focused on the relationship between public transport use and acquisition of acute respiratory infection (ARI). That study is closer to our work because it uses a more microscopical analysis, both in time and space. Data of ARI and control of other non respiratory conditions were collected from General Practitioners, together with data on bus or tram usage in the five days preceding illness onset (cases) or the five days before consultation (controls) and results show statistically significant association between ARI and bus or tram use. Moreover, other studies have also highlighted the importance of studying metropolitan patterns and social interactions. In [37], using travel smart card data, authors construct a time-resolved in-vehicle social encounter network on public buses in a city and draw attention to the impact of collective regularities can make on various diffusion/spreading processes. Our work, while addressing a similar question, is differentiate by the use of a novel analytical method combining individual-level pedestrian modelling and compartmental modelling. Moreover the two studies [36, 37] analyse transmission in a static setting (buses) while we address the problem in a dynamic environment of people moving inside underground stations.

The results of our study shows the existence of a correlation between the use of public transport and infectious diseases transmission. Specifically, we showed a correlation between the use of London underground and the spread of influenza-like illnesses. The model is particularly able to show this correlation in environments with high numbers of infections, capturing the fact that areas that have the highest numbers of ILI cases are also areas whose inhabitants spend more time in the underground network by changing line more frequently and getting in contact with more individuals. Correlation, however, does not prove causation, when looking at an epidemic on a large scale many contributing factors need to be taken into account. The role of social inequalities and age in the transmission of infectious diseases is well known and publicly accepted [38–41]. We compared the correlation between ILI cases and contacts originated from the use of the underground with the correlation coefficients between ILI cases and some demographic factors such as children and elderly populations, boroughs inner densities, employment rates and population on low income support (benefit claimants) and our results show similar values. While these results are not enough to quantify the role played by the use of public transport on large-scale infection transmission, they are interesting for both researchers and policy makers alike.

Limitations of this work are associated to the nature of the datasets involved. On the modelling side, Oyster card data and train tracking data were sourced in different years (2009 and 2013 respectively) and, while the underground network did not undergo major transformations during those years, results could still be affected by a level on uncertainty on the timetables. Also, PHE dataset reports numbers of ILI cases disaggregated only by borough and limited to one season, highly limiting the model’s precision in the evaluation of the correlation between infections and numbers of contacts. Moreover, knowledge of specific ILI-related data such as lengths of incubation periods or different level of infectiousness provided by the different pathogens causing ILI, would make results more precise and case specific. Further studies should focus, in particular, on sourcing individual-level ILI data in different settings so that, by combining them with already existing studies from household and schools, a clearer map of the transmission of ILI in a metropolitan environment could be drawn, this could help quantifying the role that different hot-spots play in the transmission. Empirical studies combining aero-biology and pedestrian modelling would be important in highly improving models fidelity and devising non-pharmaceutical control strategies tackling threshold densities to minimise numbers of infections and optimal ventilation in different crowded environments.

Policy makers, in particular, should address the role potentially played by public transport and crowded events and avoid encouraging the attendance of such environments during epidemics in order to maintain public morale, as specifically done by the *UK Influenza Pandemic Preparedness Strategy* [1].

## Conclusions

In summary, we have analysed the association between the use of public transport and infectious diseases transmission by studying the London Underground network. We used real trips data to infer the level of density in each station at any time during the day and the number of contacts between passengers, and compared these results to influenza-like illnesses (ILI) rates in London boroughs. Results show a correlation between the use of the underground and ILI cases in London, specifically they show that higher numbers of ILI cases arise in those boroughs where the population spend more time in the Underground and/or incur in a higher number of contacts when travelling. On the other hand, lower numbers of ILI cases arise in those boroughs where people have a limited use of the underground and/or incur in fewer contacts. These results are in line with other environmental and demographic factors such as population stratified by ages, inner densities employment and income.

These results are informative for both scientists and policy makers alike. At the basic research stage, further studies are required to explicitly quantify the role of public transport in infectious disease transmission, and policies should be re-evaluated to take these results into account.
